# An integrated framework of TOE, RBV, and institutional theory for understanding blockchain adoption in Egyptian public hospitals

**DOI:** 10.1038/s41598-026-57534-x

**Published:** 2026-06-27

**Authors:** Ashraf Abdou, Basma E. El-Demerdash, Sherif Mazen, Nagy Ramadan Darwish

**Affiliations:** 1 Department of Information Systems and Technology, Faculty of Graduate Studies for Statistical Research, Cairo University, Cairo, Egypt; 2 Department of Operations Research and Management, Faculty of Graduate Studies for Statistical Research, Cairo University, Cairo, Egypt; 3 Department of Information Systems, Faculty of Computers and Artificial Intelligence, Cairo University, Cairo, Egypt

**Keywords:** Blockchain, Universal Health Insurance, Adoption intention, Technology–Organization–Environment (TOE), Perceived trust, Public hospitals, Resource-based view (RBV), Institutional theory, Egypt, Health care, Health services

## Abstract

Blockchain has gained attention for its potential to verify health insurance records, allow secure data sharing, and protect patient privacy. Despite these benefits, blockchain adoption in Egypt remains limited, and little research has examined its drivers and barriers. This study contributes to the limited research on blockchain adoption in Egyptian public hospitals by extending the TOE model with context-specific factors and examining adoption decisions at the organizational level. The study was conducted in public hospitals operating within the Universal Health Insurance System (UHIS) across six governorates. A quantitative approach was used, with data collected from a stratified random sample of 228 senior management and IT professionals across 53 public hospitals, analyzed using PLS-SEM. The results show that relative advantage, financial capability, and perceived trust positively affect adoption intention, whereas perceived risk and complexity negatively affect it. Security and privacy, as well as government support and regulations, significantly enhance perceived trust but do not directly affect adoption intention. Top management support and hospital readiness also strengthen perceived trust. The model explains 67.1% of the variance in blockchain adoption intention (R^2^ = 0.671) and shows predictive relevance (Q^2^ = 0.662). Perceived trust positively influences adoption intention and mediates selected relationships in the model, particularly for security and privacy, as well as government support and regulations, which indirectly influence adoption through trust. The proposed model provides practical insights for Egyptian public hospitals, blockchain providers, the Ministry of Health, and policymakers in designing strategies that may facilitate blockchain adoption in public hospitals in Egypt.

## Introduction

Healthcare development improves quality of life and supports economic growth. Digital technologies have improved healthcare efficiency and coordination, but they have also introduced new challenges related to data security, privacy, system integration, and governance. Public hospitals operating under Egypt’s health insurance system face challenges that affect both service efficiency and the system’s long-term sustainability^1,2^. Limited budgets restrict service quality, while the absence of clear policies and health information standards makes it difficult to share medical records across systems^3^. Complex administrative processes in public hospitals also contribute to patient dissatisfaction and slow service delivery^4–6^. In addition, false claims can lead to health insurance fraud and financial losses^7^, while outdated technologies may increase the risk of data breaches involving medical records and financial information. At the broader health insurance industry level, such challenges are estimated to cost more than $11.2 billion annually by 2030, creating financial and operational pressure on healthcare systems^7,8^.

Although Egypt has moderate levels of digital infrastructure and organizational readiness compared with some developing countries, these capabilities may still be insufficient for advanced technologies such as blockchain. This may slow blockchain adoption in public hospitals, where implementation requires strong infrastructure, technical expertise, and organizational preparedness^3,9–11^. The COVID-19 pandemic further highlighted the need for digital solutions that improve healthcare coordination while protecting sensitive patient data.

Blockchain technology has been proposed as a potential solution to these challenges. In health insurance systems, blockchain can support smart contracts that automate administrative tasks and reduce operational costs^12,13^. It can also give patients greater control over their medical data, support secure information sharing^14^, and reduce fraudulent claims through immutable record-keeping^15^. Despite these potential benefits, blockchain adoption in Egypt’s health insurance sector remains limited, and many initiatives have not progressed beyond pilot or experimental stages^12,16^. Public hospitals often hesitate to implement blockchain because of integration challenges, high costs, weak regulations, and operational concerns^17–19^.

Although global research on blockchain adoption is increasing, healthcare systems in developing countries such as Egypt remain less studied than those in developed countries^12,17,18^. Two gaps constrain current understanding. First, existing blockchain adoption models give limited attention to perceived trust as a mediating mechanism that explains how technological, organizational, and institutional factors shape adoption intention^16,20,21^. Second, much of the healthcare literature focuses on individual users, such as patients or clinicians, while giving less attention to organizational-level adoption decisions. Accordingly, this study uses the hospital organizational level as the unit of analysis and examines blockchain adoption within the Universal Health Insurance System (UHIS) and the institutional and operational context of Egyptian public hospitals.

Given these gaps, this study examines the factors associated with Egyptian public hospitals’ intention to adopt blockchain within the Universal Health Insurance System, and whether trust plays a mediating role in that decision. This study contributes to the limited research by examining this question using three combined frameworks: the Technology–Organization–Environment (TOE), Resource-Based View (RBV), and Institutional Theory^21,22^.

The TOE model provides an appropriate foundation for examining technology adoption in hospitals because it covers the three main areas that shape technology adoption decisions. On the technology side, this study examines whether blockchain’s advantages, complexity, security features, and implementation risks influence adoption decisions either directly or through perceived trust. On the organizational side, it examines whether leadership support and hospital readiness matter. On the environmental side, it looks at whether available funding and government regulations play a role, particularly in shaping adoption conditions and perceived trust.

The Resource-Based View was added to improve the study’s comprehensiveness because it helps explain why some hospitals may be more ready to adopt blockchain than others, even under similar external conditions^11,21^. It reflects whether hospitals have the financial capability, infrastructure, and skills required for blockchain implementation. Institutional Theory was also incorporated because Egyptian public hospitals do not make adoption decisions in isolation^22–24^. Government directives, Ministry of Health policies, and professional standards create institutional pressures that may shape whether hospital leadership perceives blockchain as a legitimate and acceptable technology for organizational adoption. Together, these frameworks capture technological drivers, organizational resources, and institutional pressures shaping blockchain adoption in Egyptian public hospitals.

In particular, this study examines whether security and privacy, government support, and regulations influence adoption intention indirectly by strengthening perceived trust. Survey data collected from hospitals’ IT staff and managers were used to examine these adoption factors^23–25^. Accordingly, this study addressed the following research questions:

RQ1: What factors influence Egyptian public hospitals’ intention to adopt blockchain technology for health insurance systems?

RQ2: What role does perceived trust play in influencing blockchain adoption decisions in public hospitals?

The remainder of the paper is organized as follows. In “Related work” reviews the literature and theoretical framework. In “Research model and hypotheses development” section develops the research hypotheses and conceptual model. In “Methodology” section presents the research methodology and data analysis. In “Discussion” section presents and discusses the empirical results, and “Conclusion and implications” section concludes with implications, limitations, and directions for future research.

## Related work

Public hospitals in health insurance systems rely on digital transformation to manage claims, patient records, and data exchange among stakeholders. These processes require secure, transparent, and reliable information sharing, creating a need for technologies that can improve data protection and operational efficiency^16,26^. Blockchain has attracted increasing interest in healthcare because its distributed and cryptographic features can support trusted data management across multiple stakeholders^18,27^.

In health insurance systems, blockchain can support smart contracts for claims processing, improve fraud detection, and enable secure real-time access to patient data^3,28,29^. However, blockchain adoption in Egypt remains limited, and empirical research on the factors that drive or hinder its adoption in health insurance systems is still scarce^12,19,30^. This gap has encouraged the use of organizational innovation adoption frameworks to explain how public hospitals and other organizations evaluate blockchain adoption decisions^31–33^. Among these frameworks, the Technology–Organization–Environment (TOE) framework has been widely used to investigate blockchain adoption from an organizational perspective across diverse industries^34–36^. The TOE framework has also been applied since early technology adoption studies, including electronic data interchange in small organizations^37,38^.

Empirical research continuously emphasizes the importance of compatibility, perceived relative advantage, organizational innovativeness, and top management support as significant determinants influencing adoption decisions^39,40^. For instance, research in Australia and Malaysia showed that organizations take into consideration compatibility, perceived benefits, organizational readiness, and standards uncertainty as critical factors when deciding to adopt blockchain^34,41,42^. Moreover, perceived risk has been demonstrated to substantially influence adoption intentions, either directly or via moderating effects^26,43,44^. However, prior findings regarding these technological and organizational factors remain context-dependent and may vary across institutional environments, particularly in public healthcare systems. Recent empirical studies further suggest that no single factor drives blockchain adoption on its own; technology, organizational readiness, and external conditions all play a role together. This makes the TOE framework a natural fit for studying adoption decisions in complex settings, including healthcare systems^41,42,45^. In the healthcare and health insurance context, scholars have extended the TOE model to encompass sector-specific institutional and regulatory constraints^12,19^. Studies conducted in public hospitals have highlighted the roles of organizational readiness, system complexity, secure data exchange capabilities, and top management support in shaping blockchain adoption decisions^11,15,46^. Including factors such as decision-maker innovativeness and technical competence further highlights the importance of human capabilities in supporting blockchain adoption within public healthcare facilities^16,47^.

These findings suggest that blockchain adoption in healthcare depends not only on technical feasibility but also on institutional capability and governance frameworks that shape organizational decision-making^19,39,48^. Nevertheless, empirical evidence suggests that the influence of these organizational capabilities may differ depending on institutional structures and resource conditions across healthcare systems.

Several studies have integrated TOE with complementary theoretical perspectives to explore indirect and mediated relationships. Research in the insurance and financial sectors revealed that organizational factors exert a stronger influence on behavioral intention than technological or environmental factors^49–51^. Moreover, knowledge management practices were found to significantly mediate the relationships between organizational, technological, and behavioral determinants, indicating that adoption processes are rarely linear^31,49^. Similar mediation effects have been observed in studies combining TOE with diffusion and resource-based view theory^18,50,52^.

Readiness-oriented studies conducted in African healthcare and insurance systems focused on organizational and environmental preparedness rather than direct adoption intention^18,19^. Conceptual models integrating TOE with diffusion-of-innovation theory identified relative advantage, compatibility, data privacy, and cybersecurity as dominant readiness factors^21,33,52^. Environmental factors, including regulatory support, partnerships, blockchain expertise, and stakeholder engagement, have been linked to lower adoption resistance and stronger institutional readiness, although their direct influence on adoption may differ across contexts^18,39,53^. However, empirical studies report inconsistent results regarding the direct influence of regulatory and institutional pressures on blockchain adoption across countries and sectors^34,54–56^.

Beyond TOE-based approaches, alternative frameworks such as the Technology Acceptance Model (TAM), Actor Network Theory, and institutional theory have been applied to investigate blockchain adoption barriers ^18,57,58^. This research highlighted practical challenges such as insufficient budgets, insufficient knowledge of blockchain benefits, and absent governance frameworks and regulations for data sharing^18,57,59^. Therefore, the recommendations emphasized the importance of developing policies and laws alongside building institutional capacity to accelerate the adoption of blockchain in healthcare and insurance sectors^12,39,46^.

On the other hand, some studies modified the TOE model by integrating mediating factors, such as cultural compatibility, perceived privacy, perceived security, trust, perceived usefulness, or ease of use of technology^20,60,61^. Empirical findings indicate that factors such as relative advantage, competition intensity, and top management support frequently demonstrate significant effects^34,39,60^. However, other factors, such as vendor support or perceived usefulness, produce inconsistent results across contexts^20,49,60^. In other instances, mediating constructs helped explain the relationship between blockchain adoption and operational or behavioral outcomes^31,60,62^.

Comparative evidence shows that the effects of TOE factors differ across countries and sectors. Some studies focus on the importance of government regulation and institutional pressure in promoting the use of blockchain in some contexts^34,54,63^ while others report weak or non-significant direct effects^55,56^. These disparities suggest the presence of another effect that may be resulting from unobserved mediation or moderating mechanisms and demonstrate that causal relationships among adoption determinants differ significantly across organizational and national contexts^53,64^.

Importantly, previous studies have insufficiently examined the role of mediating variables in blockchain adoption models. Methodological research indicates that neglecting the role of mediation might lead to inaccurate results in interpretations of causal relationships^65,66^. Additionally, several studies neglect to include context-specific organizational and social aspects, such as trust, financial capabilities, and institutional legitimacy, which are especially pertinent in developing countries^21,61,62^.

Research on blockchain adoption remains heavily concentrated in developed economies due to their advanced infrastructure, regulatory maturity, and strong ecosystems that support innovation^26,67,68^. In contrast, empirical research concentrating on developing countries, such as Egypt, is notably limited^12,19^. The limited studies available in Egypt focus mainly on technology adoption in banking and mobile health, rather than blockchain in public hospitals^69–71^. Current evidence suggests that factors influencing adoption vary across countries due to the variations in legislative frameworks, institutional capability, economic situations, and social trust^34,54^. Thus, findings from developed country contexts may not be directly applicable to public hospitals in developing economies^21,23,64^.

Moreover, the majority of blockchain adoption studies in healthcare focus on blockchain’s technical characteristics and whether users will accept it or not, without considering the organizational and environmental factors that influence its implementation^20,24,72^. On the other hand, numerous studies analyze the factors affecting adoption from individual stakeholder perspectives, such as patients and healthcare providers, while neglecting organizational and environmental factors. Consequently, there is a lack of detailed models or frameworks for predicting how organizations, like public hospitals in Egypt, will adopt blockchain technology^39,44,49^.

Several studies emphasize the significance of contextual and moderating factors in influencing blockchain adoption decisions in addition to TOE core determinants. Cultural compatibility has been demonstrated to influence organizational adoption decisions, whereas patient-centered perspectives highlight the significance of perceived value and trust factors in a sensitive healthcare sector to keep health information secure during the exchange of patient data^21,50,60^. Cross-sector evidence also shows that blockchain adoption for risk management differs across industries, as each sector faces its own needs and challenges^67,73^. This variation highlights the need to include sectoral and contextual differences when generalizing adoption models^74^.

In summary, previous blockchain adoption research reveals three main gaps. First, most studies have focused on developed countries, leaving limited evidence from developing contexts such as Egypt^12,19,64^. Second, existing studies have paid insufficient attention to mediation mechanisms, particularly the role of perceived trust, and have not fully incorporated context-specific factors such as financial capabilities and institutional support^31,61,65^. Third, much of the healthcare literature has examined individual-level adoption perspectives, including those of doctors, nurses, and IT staff, while giving less attention to organizational and environmental conditions^12,16,44^.

These gaps indicate the need for an integrated adoption model tailored to public hospitals in resource-constrained settings. Accordingly, this study considers organizational factors, including top management support and hospital readiness, and environmental factors, including financial capabilities, government support, and regulatory conditions, to examine both direct adoption pathways and trust-related indirect pathways.

The proposed model tests both direct and mediated pathways for key technological, organizational, and environmental factors (H1–H5, H6–H9, H10–H13, H14, H15) to examine both direct relationships to adoption and indirect paths through perceived trust. This approach helps test whether trust mediates the relationship between these factors and adoption intention, which previous studies have not fully examined^31,39,61^.

## Research model and hypotheses development

A theoretical model was developed to examine the factors affecting the intention to adopt blockchain technology within the health insurance system. This section presents the proposed model, the factors identified through expert input and literature review, and the hypotheses developed to test their influence on blockchain adoption in Egyptian public hospitals. The hypotheses were derived from existing literature and adjusted to fit the Egyptian public hospital context.

### Theoretical models and hypotheses

This study integrates three theories. First, the TOE framework, developed by^75^, explains organizational technology adoption through three contextual dimensions. The technological context covers the features of the technology being considered. The organizational context includes internal resources, skills, and leadership. The environmental context refers to external forces such as regulations, market competition, and industry pressures^76^. The TOE framework has been widely applied across different sectors, including blockchain adoption in banking^77,78^, maritime industries^62^, supply chain logistics^52,79^, tourism^80^, and higher education^33^, supporting its relevance and adaptability for studying complex adoption decisions at the organizational level.

The Resource-Based View (RBV) theory, introduced by^21^, focuses on how internal resources shape an organization’s readiness to adopt new technology. The theory focuses on three elements: resources such as IT systems and digital skills, the capabilities to use them effectively, and the competitive advantage that comes from using them better than others^81^. Together, these determine whether an organization is ready to adopt and sustain new technology^81^. RBV helps explain why some hospitals are ready to adopt blockchain while others are not, even when they face the same external conditions. Hospital readiness in this study reflects RBV by capturing whether internal resources and capabilities are sufficient to implement and sustain blockchain technology^11,21^. However, in public-sector and developing-country contexts, readiness may show that an organization has the capability, but it does not always mean it has the authority or ability to implement the technology.

Institutional Theory (INS), developed by^22^, explains that organizations are influenced by external pressures when making adoption decisions, not only by internal factors. Three mechanisms explain this: mimetic pressure, where organizations follow peers to reduce uncertainty; coercive pressure, driven by government laws and regulations; and normative pressure, arising from professional standards and expectations^63,64,68^.

In the context of Egyptian public hospitals, government regulations and professional healthcare standards create institutional pressures that shape adoption conditions and perceived trust, rather than directly determining adoption decisions. In this study’s model, government support and regulations represent coercive pressure, while top management support reflects alignment with institutional expectations^23,25,99^.

Each theory used in this study has strengths and limitations. TOE is broad and covers many adoption factors, but does not deeply explain the role of culture or institutional forces. RBV theory focuses on internal resources and capabilities but tends to ignore external regulations and pressures. INS theory addresses external forces but does not account for technology-specific characteristics that shape adoption. Combining TOE, RBV, and INS with perceived trust as a mediator helps capture technological, organizational, resource-based, and institutional conditions associated with blockchain adoption in Egyptian public hospitals. At the same time, previous studies show that institutional pressures may affect organizations differently across contexts and may not always lead to actual implementation.

An extensive review of the existing literature on blockchain adoption was conducted to identify the key constructs for the proposed integrated research model. This review highlights the wide range of factors explored in previous studies across various sectors, including healthcare, supply chain, finance, and public services^16,39,49^.

Table 1 compares the constructs used in this study with those identified in prior blockchain adoption studies across different sectors. The matrix reveals two clear research gaps. Most prior studies focused on specific sectors or relied on a single theoretical framework, with few adopting a broader multi-theory perspective. Additionally, the factors influencing blockchain adoption in Egyptian health insurance systems remain largely unexplored. In this study, the unit of analysis is public hospitals, while the health insurance system represents the institutional context within which adoption decisions are made.

**Table 1:** Mapping matrix of the constructs in blockchain adoption models from previous studies.

References	Theory/model	Sector	RA	SP	PC	PR	TMS	HR	FC	GSR	PT	INS	RBV
^45^	TOE	Healthcare			✓	✓		✓	✓	✓	✓		✓
^82^	TOE/FVM/INS	Healthcare Supply chain		✓	✓	✓	✓	✓		✓	✓	✓	
^83^	TOE	Supply chain		✓	✓					✓	✓		
^84^	TOE	Supply chain	✓				✓	✓		✓	✓		✓
^78^	TOE	Banking								✓			
^63^	TOE	Multiple sectors	✓	✓			✓		✓	✓	✓		✓
^85^	TOE	Higher education	✓	✓	✓				✓				
^86^	TOE	Large enterprises	✓		✓				✓				
^87^	TAM/TOE	Quality infrastructure							✓				✓
^32^	DOI/TOE	Supply chain	✓		✓								
^88^	TOE/RBV	Construction			✓					✓			✓
^89^	Trust/TOE	Accounting	✓		✓			✓		✓			
^90^	TOE	Freight logistics	✓		✓		✓	✓	✓	✓			
^91^	TOE	Public sectors	✓		✓		✓	✓	✓	✓			
^92^	TOE/Trust	Financial			✓		✓		✓		✓		
^93^	TOE	Ecosystem	✓						✓	✓			
^94^	TOE	Supply chain	✓		✓					✓			
^95^	TOE	Supply Chain/markets	✓	✓	✓	✓			✓	✓			
^26^	TOE extended	Organizations	✓	✓		✓	✓			✓	✓	✓	
^96^	TOE	Healthcare	✓	✓		✓	✓	✓	✓	✓			
^97^	TOE	Fintech MSMEs	✓	✓	✓		✓	✓		✓	✓		
^98^	TOE	Tourism	✓		✓			✓		✓			
This Study	TOE/RBV/INS	Healthcare	✓	✓	✓	✓	✓	✓	✓	✓	✓	✓	✓

Each construct was reviewed by experts for relevance, clarity, and fit with the Egyptian public hospital context, supporting content validity^100^. The constructs are outlined in Table 4.

**Fig. 1:**
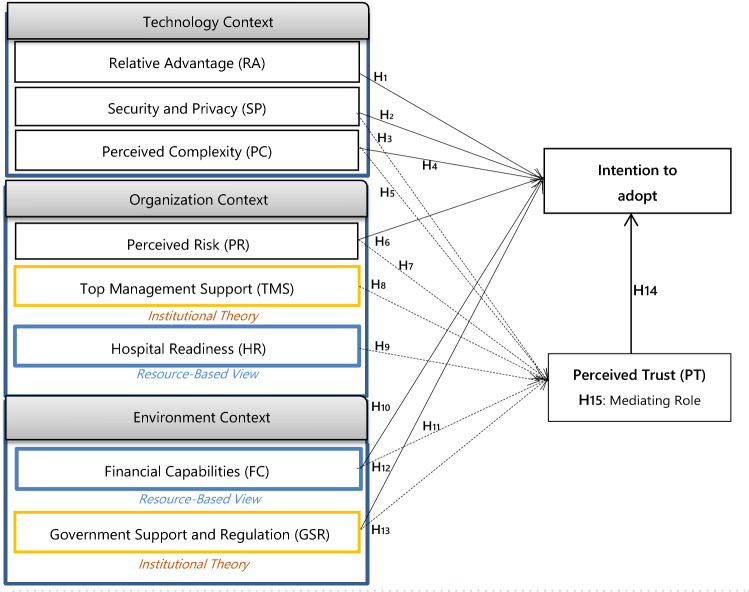
Proposed model.

The proposed model, illustrated in Fig. 1, integrates TOE model factors related to both INS and RBV theories. This study’s integrative approach provides a broader perspective of the factors influencing BCT adoption in the health insurance sector. In addition to TOE factors, this study examines the mediating role of perceived trust, analyzing its role in the relationships between TOE-related factors and adoption intention. Based on the literature review, the following hypotheses are proposed. While some factors are hypothesized to have direct effects, others may influence adoption primarily through mediating mechanisms such as perceived trust, which is tested empirically in this study. These hypotheses are proposed for empirical testing and are not assumed to be significant for all relationships.

#### H1

Relative advantage influences public hospitals’ intention to adopt BCT.

#### H2

Security and privacy influence public hospitals’ intention to adopt BCT.

#### H3

Security and privacy influence public hospitals’ trust in BCT adoption.

#### H4

Perceived complexity negatively influences public hospitals’ intention to adopt BCT.

#### H5

Perceived complexity negatively influences public hospitals’ perceived trust in BCT adoption.

#### H6

Perceived risk negatively influences public hospitals’ intention to adopt BCT.

#### H7

Perceived risk negatively influences public hospitals’ perceived trust in BCT adoption.

#### H8

Top management support influences public hospitals’ perceived trust in BCT adoption.

#### H9

Hospital readiness positively influences trust in BCT adoption.

#### H10

Financial capability influences public hospitals’ intention to adopt BCT.

#### H11

Financial capability influences public hospitals’ perceived trust in BCT adoption.

#### H12

Government support and regulations influence public hospitals’ intention to adopt BCT.

#### H13

Government support and regulations influence public hospitals’ perceived trust in BCT adoption.

#### H14

Perceived trust influences public hospitals’ intention to adopt BCT.

#### H15

Perceived trust mediates selected relationships between TOE-related factors and public hospitals’ intention to adopt BCT.

### Relative advantages (RA)

Relative advantage refers to how much an innovation improves on existing alternatives^75,76,101^. In health insurance hospitals, blockchain may offer advantages in data security and data integrity, and process transparency^8,13,102^. In the context of blockchain adoption in health insurance, relative advantage refers to how public hospitals benefit from blockchain technology through its ability to automate claims processing in insurance systems and reduce administrative expenses using smart contracts. Blockchain may provide a faster and more efficient workflow in the health insurance business cycle^8,103^. Moreover, blockchain technology has relative advantages through its core properties, such as security, transparency, and efficiency. In addition, blockchain can enable secure data sharing between insurance companies and providers, which reduces disputes and increases trust among health insurance stakeholders^21^. Based on the previous discussions, the following were assumed:

#### H1

Relative advantage influences public hospitals’ intention to adopt BCT.

### Security and privacy (SP)

Blockchain can help protect patient data by applying strong encryption algorithms to enhance privacy and security. One of the advantages of blockchain is that patient data in the insurance system will be protected from unauthorized access by third parties, and the patients may gain greater control over their data^14,104^. Moreover, blockchain enables hospitals to manage access to sensitive patient data effectively^105,106^. These characteristics may increase trust and collaboration among health insurance stakeholders, including insurers, providers, and patients. They may also reduce administrative complexity in health insurance processes, supporting blockchain adoption by hospitals^107^. Therefore, security and privacy are examined for both their direct association with adoption intention and their indirect role through perceived trust. Therefore, the hypotheses are proposed:

#### H2

Security and privacy influence public hospitals’ intention to adopt BCT.

#### H3

Security and privacy positively influence public hospitals’ trust in BCT adoption.

### Perceived complexity (PC)

Perceived complexity refers to how individuals or organizations assess the potential difficulties and challenges related to adopting new technology^108^. In general, the more complex or difficult an innovation is to understand or use, the lower the likelihood of its adoption and successful implementation^109^.

Complexity may appear in several ways, such as difficulty understanding blockchain technology, using it, or integrating it smoothly into existing systems. It includes technical challenges, scalability issues, the need for particular expertise, and the difficulty of integrating blockchain with existing infrastructure^58^.

Also, if the technology has a high level of complexity, it can create uncertainty for organizations when making adoption decisions. If organizations find that the risks and costs of implementing the technology outweigh the expected benefits, adoption intention decreases. Based on the above, we proposed that:

#### H4

Perceived complexity negatively influences public hospitals’ intention to adopt BCT.

#### H5

Perceived complexity negatively influences public hospitals’ perceived trust in BCT adoption.

Figure 1 presents the integrated research model for blockchain adoption. The model combines the TOE framework with Institutional Theory (orange-bordered boxes) and Resource-Based View (blue-bordered boxes). Solid arrows represent direct hypothesized relationships (H1–H14). Dashed arrows represent indirect relationships through Perceived Trust (PT) as a mediating variable (H15a–g).

### Perceived risk (PR)

Perceived risk is defined as the negative impact that an organization expects to have if it intends to use a new technology^74,110^. If public hospitals in Egypt do not have the required skills (i.e., technical expertise and practical knowledge) to implement, operate, and maintain blockchain technology in health insurance systems. In that case, they are unlikely to adopt blockchain. The technical complexity and difficulty of integrating with existing systems are risks facing blockchain adoption. In addition, risks stem from the high costs associated with implementation, establishing a robust infrastructure, and ongoing maintenance^43,44^.

Perceived trust stems from organizational culture and influences technology adoption. In some contexts, perceived risk may inversely relate to trust when organizations perceive high implementation risks, confidence in technology adoption may decrease^61^. Public hospitals are hesitant to adopt new technology for health insurance systems, especially in a sensitive sector like health insurance, when there are no pilot projects demonstrating the success of blockchain implementation in similar settings.

Institutional theory suggests that uncertainty and lack of proven examples may weaken organizational confidence in new technologies.

Furthermore, if the organization has a shortage of required professional resources in hospitals, and decision-makers are uncertain about the return on investment from adopting blockchain technology, these factors can create uncertainty in adopting the technology^111^. Accordingly, we suggest the following:

#### H6

Perceived risk negatively influences public hospitals’ intention to adopt BCT.

#### H7

Perceived risk negatively influences public hospitals’ perceived trust in BCT adoption.

### Top management support (TMS)

Top management is responsible for making technology adoption decisions; they are the decision-makers accountable for adopting innovative technologies and overseeing their implementation. Adopting blockchain technology is a strategic decision and requires high financial investments. Moreover, blockchain adoption in health insurance is costly, and it requires the readiness of hospitals in terms of infrastructure. For that, the decision to adopt needs significant support from top management, which plays a key role in building organizational trust. Top management can provide the necessary resources and build trust by demonstrating a commitment to implementation within the organization. Such actions may reduce resistance and increase confidence in implementation^25,108^. Also, the support of top management will assist in lowering the level of opposition to adopting blockchain technology^76^. Accordingly, the following hypothesis is proposed:

#### H8

Top management support influences public hospitals’ perceived trust in BCT adoption.

### Hospital readiness (HR)

Egyptian public hospitals need robust IT infrastructure, dedicated budgets for digital transformation, and compatibility with existing data management systems^11^. Prior research indicates that organizational readiness regarding resources, technical competencies, and infrastructure influences the trust and confidence that decision-makers have in the viability of technology adoption. When hospitals show that they are ready to use technology, decision-makers become more confident in making an adoption decision^112^. Thus, we proposed the following hypothesis:

#### H9

Hospital readiness positively influences trust in BCT adoption.

### Financial capability (FC)

The Egyptian government should have a budget allocated to blockchain application development and its continuous maintenance and optimization to ensure long-term efficiency. Financial capabilities represent the level of budgetary resources available to the Egyptian health sector to function effectively^81,113^.

Financial capabilities comprise the hospital’s capacity to provide sufficient funds to implement technology in infrastructure, training, and continuous maintenance of blockchain-based systems^114^. Financial capabilities also include the availability of external funds (e.g., government grants and funding from external donors), the government’s ability to make strategic financial investments, and the provision of a special government budget for investing in technology^34^.

The availability of a financial budget allocated to support technology in public hospitals will increase decision-makers’ confidence in adopting blockchain. Such confidence grows when the cost of training, developing, and building support systems to implement blockchain applications is available^115^. Accordingly, this study lists these hypotheses:

#### H10

Financial capability influences public hospitals’ intention to adopt BCT.

#### H11

Financial capability influences public hospitals’ perceived trust in BCT adoption.

### Government support and regulations (GSR)

Government support may create favorable conditions for blockchain adoption by strengthening perceived trust, legitimacy, and implementation readiness^54,116^. Regulations and policies may create a sense of reliability and legitimacy that increases top management trust in blockchain adoption^23,63,109^. Data exchange protocol and encryption standards are among the most important regulations that must be followed to secure patient data. However, regulatory support’s direct effect on adoption depends on whether policies include practical implementation resources and guidance. In this context, government support and regulations are expected to influence adoption indirectly through perceived trust rather than directly affecting adoption intention. Based on this discussion, we assume the following:

#### H12

Government support and regulations influence public hospitals’ intention to adopt BCT.

#### H13

Government support and regulations influence public hospitals’ perceived trust in BCT adoption.

### Perceived trust (PT)

Perceived trust in technology means the confidence that the technology will perform consistently and safely, especially in uncertain or risky conditions^43,117^. Many previous studies have explored the role of trust across various domains, including supply chain management, digital payments, and e-commerce^118,119^.

Thus, trust is important to overcome risks and threats associated with handling sensitive personal and medical data. Trust in blockchain’s ability to protect this data is crucial^120^. The cryptographic feature of blockchain provides it with higher transparency in transactions, e.g., insurance claims can be easily tracked, and stakeholders can view and track the history of claims, which augments the trust in the technology^121^. Therefore, we could hypothesize the following:

#### H14

Perceived trust influences public hospitals’ intention to adopt BCT.

### The mediating role of perceived trust

Previous studies have extensively examined the role of trust in technology adoption contexts, employing trust as a mediating factor combined with various theoretical models, including TAM, DOI, UTAUT, and TOE, which provide insight into how trust affects the use of technology^92,122–124^. Recent studies confirm that trust mediates the relationship between perceived usefulness and ease of use^125^. Also, it serves as a key mediator between system characteristics and behavioral responses^126^. On the other hand, blockchain-based trust models have been proposed for healthcare IoT systems^127^.

Perceived trust was included in this study because of its role in ensuring the protection of sensitive patient information. The absence of a broader legislative framework for technology adoption in Egypt contributes to ambiguity. Also, the medical and administrative departments in public hospitals have a resistance to change and a need for trust to adopt blockchain.

Including trust as a mediating variable was essential to assess its influence on technology acceptance and to investigate its correlations with TOE factors. The mediating role of trust is examined empirically rather than assumed across all hypothesized relationships. For that, this hypothesis is proposed:

#### H15

Perceived trust mediates selected relationships between TOE-related factors and public hospitals’ intention to adopt BCT.

## Methodology

### Research approach

This study utilized a positivist research methodology, focusing on quantitative analysis and hypothesis testing. A cross-sectional questionnaire design was used to examine technological, organizational, and environmental factors, perceived trust as a mediator, and their direct and indirect effects on blockchain adoption intention^61,108,113,117,128^. The survey method has been used to systematically assess participants’ preferences and decisions regarding blockchain adoption^129^. The questionnaire measured ten reflective constructs using 43 indicators adapted from prior studies to fit Egypt’s health insurance context (Table 4). A survey approach was chosen because it allows statistical testing of relationships among multiple constructs and hypotheses to be tested at the organizational level. Responses were collected from hospital IT professionals, managers, and relevant decision-makers working in public hospitals, and the analysis examines adoption decisions at the hospital level within the UHIS institutional context.

The model was validated by five experts from academia, technology, and healthcare, who assessed each construct for relevance, clarity, and fit with the Egyptian context. Their feedback led to revisions that improved questionnaire clarity, and plain language was used throughout to ensure participants understood each question. Such expert review strengthened the model’s content validity and credibility^22^.

A pilot study was conducted to confirm questionnaire reliability and refine unclear items before full data collection.

### Sampling and population

The broader study context included public healthcare facilities operating under the first phase of Egypt’s Universal Health Insurance System across six governorates. These facilities comprised 53 public hospitals and 295 family medicine units and centers. However, because the unit of analysis was the hospital level, the empirical sample was drawn only from personnel working in the 53 public hospitals, where organizational adoption decisions are made. Stratified random sampling was applied to account for the uneven distribution of public hospitals across governorates.

The target population was divided into similar groups for each governorate, and samples were selected proportionally to minimize bias and ensure the samples represent the population^76,95,130^. Based on the sampling rules set out by^131^, and established sample size guidelines^132^, a target sample size of 300 was suitable for the study, depending on the total number of populations. Table 2 shows how hospitals and healthcare facilities are distributed and how the samples are divided across the chosen governorates.

**Table 2:** Stratified sampling distribution and response rates.

Governorate	Hospitals	Family medicine units	Family medicine centers	Total population	Sample	Percentage (out of 348)	Response	Governorate
Port Said	11	28	7	46	35	10.05	26	8.6
Ismailia	12	30	9	51	45	12.9	36	12
Luxor	7	50	9	66	60	17.2	44	14.6
South Sinai	8	18	5	31	25	7.18	16	5.3
Aswan	11	100	12	123	110	31.6	88	29.3
Suez	4	22	5	31	25	7.18	18	6
Total	53	248	47	348	300	86.20	228	76

Of the 300 questionnaires distributed to participants at the 53 public hospitals between January and June 2024, 250 responses were received. Of these, 22 were identified as duplicates and removed, leaving 228 valid responses and a final response rate of 76%, which exceeds typical online survey benchmarks^133^.

### Data collection

Questionnaires were sent by email to participants, and some of the data was collected by conducting in-person interviews and utilizing Google Forms. Online survey methods offer benefits for organizational research^134,135^. Participation invitations were sent to top managers to disseminate the questionnaire to IT employees, hospital administrators, IT directors, and leaders at the strategic level^119,136^.

There were two parts to the questionnaire. The first part asked the respondents to fill in some demographic data related to their gender, age, job title, level of education, and level of understanding about blockchain, as shown in Table 3.

**Table 3:** Demographic characteristics of respondents.

Category	Item	Frequency	Percentage
Gender	Male	156	68.4
	Female	72	31.6
Age	18–31 years	14	6.1
	32–35 years	81	35.5
	36–41 years	77	33.8
	42–46 years	46	20.2
	47–55 years	5	2.2
	Over 55 years	5	2.2
Job role	Head of information systems	5	2.2
	Lead of medical IT executive	26	11.4
	Technology strategy director	27	11.8
	Hospital operations manager	25	11.0
	Department director	7	3.1
	IT directors	133	58.3
	Senior clinicians	5	2.2
Educational level	High school	11	4.8
	Bachelor	41	18.0
	Diploma	16	7.0
	Master’s degree	160	70.2
Blockchain knowledge	Poor	148	65.0
	Moderate	46	20.2
	Good	22	9.6
	Very good	12	5.2

The second section consisted of 43 measurement items related to 10 constructs within the proposed research model. Table 4 shows the constructs’ definitions and the items used to measure them. A five-point Likert scale, with 1 being “strongly disagree” and 5 being "strongly agree," was used to rate all of the measurement items. The midpoint represented a neutral response^107^.

**Table 4:** List of constructs and measurement Items.

Construct	Code	Measuring items	References
Relative advantage (RA)	RA_1_	Blockchain enhances healthcare efficiency by streamlining processes	^68,59,116^
	RA_2_	Blockchain reduces administrative burdens by automating claims processing	
	RA_3_	Blockchain improves transparency and traceability in health insurance systems	
	RA_4_	Blockchain adoption can reduce operational costs in health insurance processes	
	RA_5_	Blockchain enables interoperability and data sharing among healthcare providers and insurers	
	RA_6_	Blockchain enables healthcare systems to operate more efficiently by ensuring accurate data and timely access	
	RA_7_	Blockchain can support scalability in national health insurance programs	
Security and privacy (SP)	SP_1_	Blockchain ensures data security and patient confidentiality	^14,105,106^
	SP_2_	Blockchain enhances healthcare data protection through advanced encryption algorithms	
	SP_3_	Blockchain enables patients to have greater control over their data while supporting privacy compliance	
Perceived complexity (PC)	PC_1_	Blockchain is difficult for hospital staff to understand	^108,109,121^
	PC_2_	Integrating blockchain with healthcare systems is challenging	
	PC_3_	Setting up a blockchain requires significant time and effort	
	PC_4_	Blockchain adoption requires specialized skills that are limited in healthcare organizations	
Perceived risk (PR)	PR_1_	Hospital employees may resist blockchain due to uncertainty or a lack of familiarity	^108,109,121^
	PR_2_	Public hospitals in Egypt lack the expertise to implement and manage blockchain	
	PR_3_	Integrating blockchain with legacy systems poses some technical and organizational risks	
	PR_4_	The lack of blockchain pilot projects creates uncertainty about its feasibility in healthcare	
Top management support (TMS)	TMS_1_	Top management allocates sufficient resources for blockchain adoption in public hospitals	^152,121^
	TMS_2_	The top management has a clear vision for blockchain implementation	
	TMS_3_	Top management supports collaboration with health insurance stakeholders to mitigate blockchain adoption risks	
Hospital readiness (HR)	HR_1_	The hospital has adequate IT infrastructure for blockchain implementation	^11,31,129^
	HR_2_	The hospital has sufficient internal resources to support the implementation of blockchain	
	HR_3_	The hospital’s internal business workflows are adaptable to blockchain	
Financial capabilities (FC)	FC_1_	External funding, including grants and aid, supports blockchain adoption in Egypt	^34,81,114^
	FC_2_	Government budget allocation supports successful technology adoption	
	FC_3_	Egypt’s economic conditions support investment in blockchain technology	
	FC_4_	Public–private investments support blockchain adoption in Egypt	
Government support and regulations (GSR)	GSR_1_	Government policies encourage hospitals to adopt blockchain	^116,121,63^
	GSR_2_	Government support provides financial backing for blockchain adoption	
	GSR_3_	Government support strengthens digital infrastructure for blockchain adoption	
	GSR_4_	The government must establish clear laws and enforce regulations for blockchain safety in healthcare	
Perceived trust (PT)	PT_1_	Blockchain is perceived as secure and reliable for managing healthcare data	^61,117–119,136,143,138^
	PT_2_	Blockchain ensures data integrity, building trust in its adoption in healthcare	
	PT_3_	The immutable nature of blockchain enhances trust in its application	
	PT_4_	Trust in organizational leadership promotes confidence in blockchain adoption	
	PT_5_	Employee trust in blockchain technology supports its implementation	
	PT_6_	Government support and regulation increase trust in blockchain adoption	
	PT_7_	Adherence to data protection laws builds trust in the use of blockchain in healthcare	
	PT_8_	Blockchain transparency increases trust among healthcare stakeholders	
Intention to use (INT)	INT_1_	Public hospitals are likely to adopt blockchain due to its advantages	^152,121^
	INT_2_	Public hospitals intend to adopt blockchain if it supports compliance with data protection laws	
	INT_3_	Public hospitals intend to adopt blockchain if it addresses data breach and regulatory compliance concerns	

Before starting and filling out the questionnaire, all volunteers were informed of the purpose of the study. The confidentiality of responses was maintained, and all participants provided informed consent. The study was approved by the Faculty of Graduate Studies for Statistical Research and conducted in accordance with relevant ethical guidelines and regulations.

### Data analysis tools

This study employed PLS-SEM using SmartPLS 4.0 to analyze survey data and evaluate the proposed hypotheses^137^. It was selected because it is suitable for complex structural models with multiple constructs and mediation paths. PLS-SEM is widely used when sample sizes are moderate and when models include multiple constructs and mediation paths. The empirical analysis was conducted on 228 valid responses collected from healthcare IT professionals working in 53 public hospitals. The analysis followed the standard two-stage PLS-SEM procedure: assessment of the measurement model followed by assessment of the structural model^117,128,138,139^.

### Variable notation and demographic profile

For analytical clarity, the study variables were defined as follows: hospitals’ intention to adopt blockchain (INT) as the dependent variable. Intention predicts actual behavior in organizational technology adoption contexts^140^. Perceived Trust (PT) was used as a mediating variable to examine its role in transmitting the effects of technological and environmental factors to adoption intention. Security and Privacy (SP), Relative Advantages (RA), and Perceived Complexity (PC) are all technological factors. Organizational factors include top management support (TMS) and hospital readiness (HR), with hospital readiness reflecting the Resource-Based View (RBV). Environmental factors include financial capabilities (FC) and government support and regulations (GSR), with GSR reflecting institutional pressures from Institutional Theory (INS).

All constructs and indicators were modified from validated instruments stated in previous studies and customized for the Egyptian public healthcare context^59,61,73,75,103,130,141–143^.

Before applying SEM, a preliminary analysis was conducted to make sure the data contained no missing values and to minimize non-response bias. Variance equality was assessed to verify statistical assumptions^144^. Diagnostic tests were performed following established procedures to confirm the data’s suitability for SEM analysis^145^.

To reduce the chances of bias, only respondents who work in the IT or health insurance sector at public hospitals were allowed to fill out the questionnaire. Such a selection ensured that responses were relevant to the study context^95,118,119^. This approach allows the study to empirically validate whether certain factors influence adoption directly or primarily through mediation mechanisms.

Table 3 shows the demographic information about the participants who answered the survey. There were 68.4% men and 31.6% women in the sample, and most of them were between the ages of 32 and 41 (69.3%). 58.3% of the respondents have an IT director title, and 70.2% held master’s degrees. About 65% of those who answered declared they didn’t have enough knowledge about blockchain technology, while 20.2% stated they knew a little about it. Only 14.8% of those who answered said they were highly familiar with blockchain. This distribution indicates limited blockchain familiarity among respondents.

### Empirical results

#### Common method bias

This section presents the results after applying PLS-SEM in two stages, including the assessment of the measurement and structural models. The analysis was done using SmartPLS 4.0, and a bootstrapping process of 5,000 subsamples was used to evaluate the proposed hypotheses.

Since respondents provided data on both dependent and independent variables, Common Method Bias (CMB) was a potential concern^146,147^. Harman’s single-factor test^148^ was used to assess this, revealing that a single factor explained a maximum variance of 32.45%, well below the 50% threshold. Such a result confirms that CMB was not a significant issue in this dataset. Collinearity was also tested using the Variance Inflation Factor (VIF). Harman’s single-factor test indicated that the largest single factor explained 32.45% of the variance, below the 50% threshold, suggesting that common method bias was not a major concern.

Collinearity was assessed separately using VIF values. As shown in Table 9, all VIF values were below 5, indicating acceptable collinearity levels for the structural model.

#### Measurement model

Fornell’s approach was used to assess discriminant validity, and the measurement model was evaluated using established factor analysis procedures before analyzing the structural relationships among constructs^149,150^. The process involved checking both the reliability and validity of study constructs. Two reliability tests have been used: Cronbach’s alpha and composite reliability (CR). Cronbach’s alpha shows how consistently the items in each construct relate to one another, while CR refers to how well the indicators capture what the construct is supposed to measure. Both values run from 0 to 1; a higher number means better reliability. In exploratory studies like this one, scores between 0.60 and 0.70 are acceptable, and anything above 0.70 is considered good. Table 5 shows that all constructs had strong internal consistency. CR values ranged from 0.790 to 0.934, and Cronbach’s alpha ranged from 0.756 to 0.908^151^.

**Table 5:** Assessments of reliability and convergent validity.

Construct	Items	VIF	Outer loadings	Cronbach’s alpha	CR	AVE
Relative advantage (RA)	RA_1_	1.765	0.743	0.851	0.887	0.533
	RA_2_	1.868	0.708			
	RA_3_	1.345	0.706			
	RA_4_	2.421	0.837			
	RA_5_	2.429	0.810			
	RA_6_	2.14	0.792			
	RA_7_	1.768	0.677			
Security and privacy (SP)	SP_1_	2.186	0.884	0.824	0.895	0.740
	SP_2_	1.758	0.863			
	SP_3_	1.829	0.831			
Perceived complexity (PC)	PC_1_	3.828	0.905	0.908	0.934	0.781
	PC_2_	2.826	0.904			
	PC_3_	2.373	0.807			
	PC_4_	3.364	0.915			
Perceived risk (PR)	PR_1_	1.892	0.804	0.839	0.891	0.673
	PR_2_	1.973	0.876			
	PR_3_	1.671	0.799			
	PR_4_	1.839	0.795			
Top management support (TMS)	TMS_1_	1.507	0.762	0.612	0.790	0.557
	TMS_2_	1.142	0.681			
	TMS_3_	1.358	0.792			
Hospital readiness (HR)	HR_1_	1.595	0.803	0.756	0.860	0.671
	HR_2_	1.750	0.850			
	HR_3_	1.387	0.610			
Financial capabilities (FC)	FC_1_	1.335	0.684	0.770	0.853	0.593
	FC_2_	1.767	0.825			
	FC_3_	1.390	0.717			
	FC_4_	1.811	0.840			
Government support and regulations (GSR)	GSR_1_	2.035	0.832	0.832	0.888	0.666
	GSR_2_	2.350	0.863			
	GSR_3_	1.727	0.799			
	GSR_4_	1.649	0.771			
Perceived trust (PT)	PT_1_	1.950	0.630	0.839	0.894	0.517
	PT_2_	1.550	0.794			
	PT_3_	1.697	0.705			
	PT_4_	2.047	0.720			
	PT_5_	1.561	0.753			
	PT_6_	2.763	0.805			
	PT_7_	2.468	0.798			
	PT_8_	2.629	0.811			
Intention to use (INT)	INT_1_	1.236	0.701	0.64	0.805	0.583
	INT_2_	1.261	0.707			
	INT_3_	1.478	0.880			

The dependability of an indicator in the model is assessed by the value of outer loadings. Such a value refers to the degree to which each item in a latent-variable model reflects the construct it is intended to represent. Typical outer loadings are 0.708 or greater. Items with loadings ranging from 0.40 to 0.70 warrant careful evaluation and are often removed. The value of outer loading will be removed only when its exclusion leads to an improvement in AVE or composite reliability values. In this study, items with loadings below 0.40 were excluded from the measurement model. As shown in Table 5, the majority of indicators exceeded the 0.70 threshold for outer loadings.

Four items had outer loadings between 0.40 and 0.70: RA7 (0.677), HR3 (0.610), PT1 (0.630), and FC1 (0.684). These items were retained because their removal did not substantially improve AVE or composite reliability values. Most Cronbach’s alpha values exceeded 0.70, while TMS and INT were slightly below this threshold. However, their composite reliability values exceeded 0.70 and AVE values exceeded 0.50, indicating acceptable reliability and convergent validity for exploratory research^153^.

Convergent validity was assessed using Average Variance Extracted (AVE). The AVE values ranged from 0.517 to 0.781, exceeding the recommended threshold of 0.50 and confirming convergent validity for all constructs (see Table 5).

Discriminant validity tests whether each construct measures something distinct from other constructs. We used two approaches: the Fornell-Larcker criterion and the heterotrait-monotrait ratio of correlations (HTMT).

The Fornell–Larcker criterion measures whether the square root of each construct’s Average Variance Extracted (AVE) is more than its correlations with other constructs. Table 6 reveals that the square root of the Average Variance Extracted (AVE) for each construct (diagonal values) exceeded its maximum correlation with any other construct, signifying sufficient discriminant validity among all constructs^149^.

**Table 6:** Discriminant validity based on Fornell–Larker criterion.

Construct	FC	GSR	HR	INT	PC	PR	PT	RA	SP	TMS
FC	0.770									
GSR	0.740	0.816								
HR	0.579	0.792	0.819							
INT	0.601	0.710	0.740	0.763						
PC	− 0.248	0.601	− 0.215	− 0.146	0.884					
PR	− 0.416	− 0.248	− 0.252	− 0.208	0.805	0.820				
PT	0.709	0.731	0.672	0.658	− 0.190	− 0.255	0.719			
RA	0.595	0.708	0.582	0.710	− 0.118	− 0.318	0.589	0.730		
SP	0.519	0.595	0.588	0.686	− 0.120	− 0.273	0.607	0.708	0.860	
TMS	0.618	0.519	0.606	0.570	− 0.265	− 0.271	0.709	0.493	0.502	0.746

The HTMT, proposed by Henseler^154^, compares the average correlations between indicators of different constructs to the geometric mean of average correlations within a single construct. HTMT values over 0.90 may suggest issues with discriminant validity, but a threshold of 0.85 is considered acceptable.

As presented in Table 7, most HTMT values were below 0.85, while a few slightly exceeded this threshold but remained below 0.90, indicating acceptable discriminant validity under the more liberal criterion^154^.

**Table 7:** Discriminant validity based on HTMT.

Construct	FC	GSR	HR	INT	PC	PR	PT	RA	SP	TMS
FC										
GSR	0.818									
HR	0.766	0.705								
INT	0.852	0.716	0.714							
PC	0.280	0.211	0.264	0.189						
PR	0.504	0.335	0.296	0.280	0.811					
PT	0.821	0.839	0.827	0.814	0.203	0.286				
RA	0.745	0.674	0.699	0.862	0.161	0.363	0.672			
SP	0.640	0.598	0.728	0.810	0.138	0.309	0.717	0.817		
TMS	0.824	0.710	0.811	0.760	0.340	0.393	0.820	0.658	0.657	

**Fig. 2:**
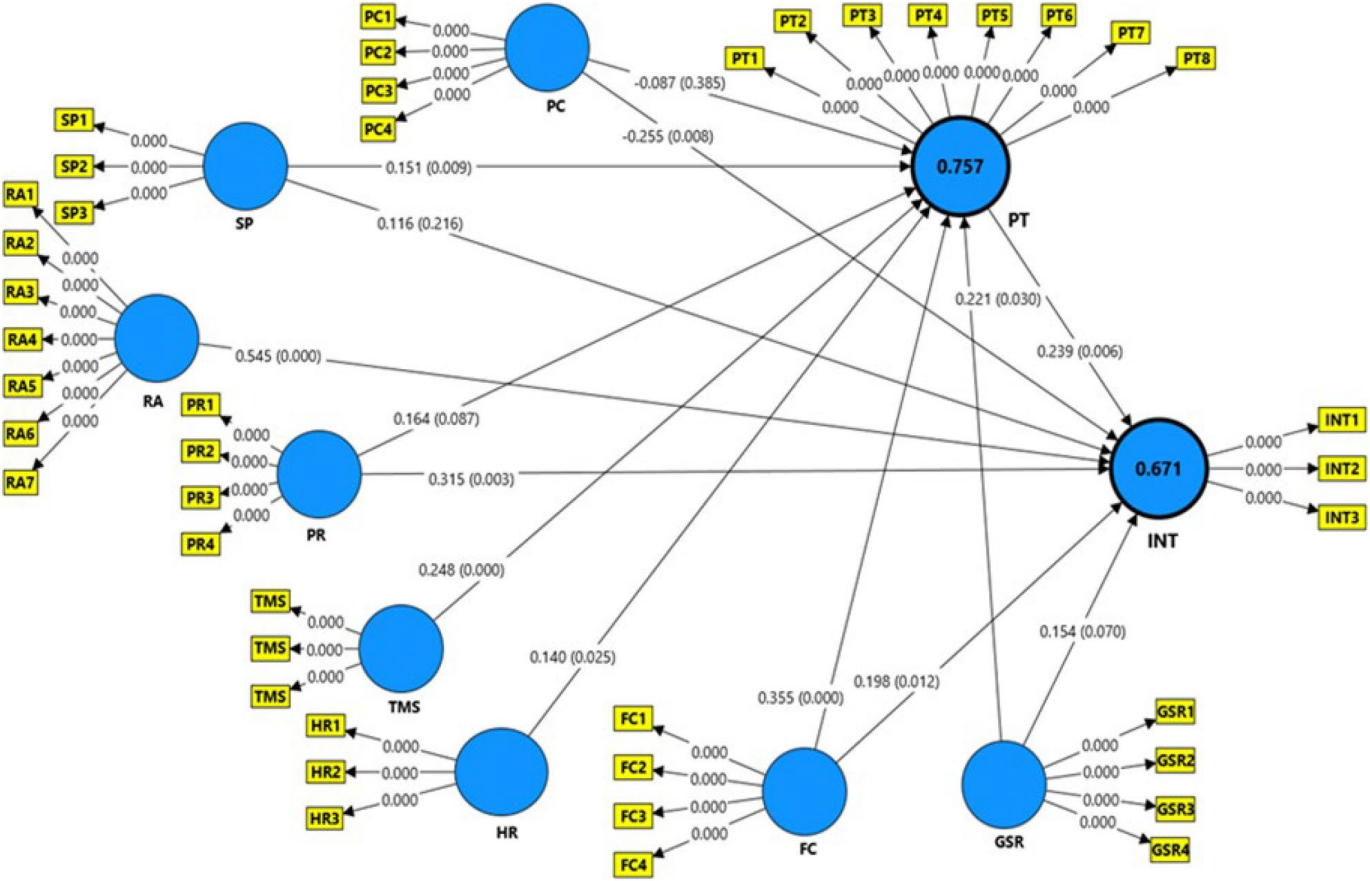
Structural model result.

Figure 2 presents the structural model results, showing the hypothesized relationships among the study constructs. The structural model was assessed through five steps: (1) verifying the adequacy of the measurement model to ensure reliable and valid construct measurement; (2) assessing multicollinearity using the Variance Inflation Factor (VIF); (3) evaluating the significance and relevance of path coefficients to test the proposed hypotheses; (4) assessing the strength and direction of relationships using path coefficients and t-statistics; and (5) evaluating the model’s explanatory and predictive power for the dependent variables.

#### Structural model measurement results and testing the research hypotheses

Table 8 displays the results of the structural relationship analysis, showing the proposed cause-and-effect relationships among the study constructs, while Fig. 2 visually represents these relationships.

**Table 8:** The analysis of the path coefficients.

Hypotheses	Structure path	Causal relationship	Path coefficients β	T-statistic (|O/STDEV|)	p-values	95% Bias		Result
						LB	UB	
Direct effect								
H_1_	RA → INT	+ VE	0.545	5.711	0.001	0.351	0.721	Accepted
H_2_	SP → INT	+ VE	0.116	1.236	0.216	− 0.058	0.309	Rejected
H_3_	SP → PT	+ VE	0.151	2.604	0.009	0.052	0.283	Accepted
H_4_	PC → INT	– VE	− 0.255	2.641	0.008	− 0.467	− 0.097	Accepted
H_5_	PC → PT	– VE	− 0.087	0.869	0.385	− 0.289	0.098	Rejected
H_6_	PR → INT	– VE	− 0.315	3.014	0.003	− 0.547	− 0.142	Accepted
H_7_	PR → PT	– VE	− 0.164	1.710	0.087	− 0.011	0.359	Rejected
H_8_	TMS → PT	+ VE	0.248	3.737	0.001	0.071	0.344	Accepted
H_9_	HR → PT	+ VE	0.140	2.240	0.025	0.013	0.262	Accepted
H_10_	FC → INT	+ VE	0.221	2.166	0.030	0.039	0.419	Accepted
H_11_	FC → PT	+ VE	0.355	4.127	0.001	0.170	0.517	Accepted
H_12_	GSR → INT	+ VE	0.154	1.811	0.070	− 0.004	0.325	Rejected
H_13_	GSR → PT	+ VE	0.198	2.524	0.012	0.047	0.354	Accepted
H_14_	PT → INT	+ VE	0.239	2.734	0.006	0.054	0.399	Accepted
Indirect effect/mediating effect								
H_15_								
H_15_ a	PC → PT → INT	– VE	− 0.021	0.788	0.431	− 0.073	0.035	Rejected
H_15_ b	PR → PT → INT	– VE	− 0.039	1.443	0.149	− 0.010	0.096	Rejected
H_15_ c	SP → PT → INT	+ VE	0.040	1.994	0.046	0.014	0.095	Accepted
H_15_ d	TMS → PT → INT	+ VE	0.059	2.084	0.037	0.007	0.119	Accepted
H_15_ e	FC → PT → INT	+ VE	0.085	2.306	0.021	0.019	0.163	Accepted
H_15f_	GSR → PT → INT	+ VE	0.055	2.273	0.023	0.015	0.264	Accepted
H_15_ g	HR → PT → INT	+ VE	0.033	1.767	0.077	− 0.004	0.080	Rejected

β and p-values for H15a–H15g represent bootstrapped indirect effects. The f^2^ effect size is reported only for direct predictor effects on endogenous constructs; therefore, f^2^ values are presented separately in Table  9.

**Table 9:** Structural model assessment.

Construct	Effect size (f-square)		Collinearity (VIF)	
	Intention to use	Perceived trust	Intention to use	Perceived trust
RA	0.254		3.561	
SP	0.012	0.052	3.294	1.814
PC	0.056	0.009	3.517	3.570
PR	0.073	0.028	4.142	3.996
TMS		0.124		2.043
HR		0.039		2.081
FC	0.035	0.138	4.202	3.776
GSR	0.024	0.056	3.039	2.877
PT	0.051		3.384	

	Intention to adopt	Perceived trust
R square	0.671	0.757
Q square	0.662	0.721

The proposed model examined each relationship with direct or indirect effects by calculating the path coefficient values (β) and T-values. Hair^139^ indicates that the path coefficients must be higher than 0.1 and the T-values must be higher than 1.64 at a significance level of 0.05. These criteria have been used to test the hypotheses. Path coefficients (β) show the relationship strength and direction between variables. P-values indicate statistical significance (typically p < 0.05). The 95% confidence interval (LB/UB) provides the range where the true coefficient likely falls, from the lower bound to the upper bound. We evaluated direct and indirect effects using bootstrapped path coefficients (β), p-values, and 95% confidence intervals, as reported in Table 8.

#### Hypotheses testing

Path coefficients (β) and their corresponding statistical significance (T-values) were evaluated through bootstrapping with 5,000 subsamples. The results were presented in Table 8. Figure 2 illustrates the path coefficients for the main TOE factors, with direct effects on intention to adopt blockchain: The data show significant positive relationships for H1 (RA → INT) was supported (β = 0.545, p = 0.001, f^2^ = 0.254) and H10 (FC → INT) was supported (β = 0.221, p = 0.030, f^2^ = 0.035), supporting these hypotheses in this sample.

Significant negative relationships were found for H4 (PC → INT), which was supported (β = -0.255, p = 0.008, f^2^ = 0.056), and H6 (PR → INT) was supported (β = -0.315, p = 0.003, f^2^ = 0.073), indicating significant negative effects of complexity and risk on adoption intention.

Two hypotheses were not supported: H2 (SP → INT) was rejected (β = 0.116, p = 0.216, f^2^ = 0.012) and H12 (GSR → INT) was rejected (β = 0.154, p = 0.070, f^2^ = 0.024). These results explicitly show that security and privacy, government support, and regulations do not have significant direct effects on adoption intention in this context.

Effects on perceived trust. The results show that several factors significantly strengthened perceived trust. H3 (SP → PT) was supported (β = 0.151, p = 0.009, f^2^ = 0.052), H8 (TMS → PT) was supported (β = 0.248, p = 0.001, f^2^ = 0.124), H9 (HR → PT) was supported (β = 0.140, p = 0.025, f^2^ = 0.039), H11 (FC → PT) was supported (β = 0.355, p = 0.001, f^2^ = 0.138), and H13 (GSR → PT) was supported (β = 0.198, p = 0.012, f^2^ = 0.056). These results indicate that security and privacy, top management support, hospital readiness, financial capability, and government support and regulations contribute to increased trust. In contrast, H5 (PC → PT) was rejected (β = -0.087, p = 0.385, f^2^ = 0.009), and H7 (PR → PT) was rejected (β = -0.164, p = 0.087, f^2^ = 0.028), indicating that perceived complexity and perceived risk did not significantly affect perceived trust in this sample.

The direct effect of H14 (PT → INT) was supported (β = 0.239, p = 0.006, f^2^ = 0.051). This result supports H14 and indicates a significant positive relationship between perceived trust and adoption intention. The mediation analysis (H15) indicated that perceived trust significantly mediates selected relationships (as outlined in Table 8), with particular indirect effects validated by using bootstrapped confidence intervals.

#### Mediation analysis

The mediating role of perceived trust was examined through H15, which tested whether selected TOE-related factors influence adoption intention indirectly through perceived trust. Indirect effects were assessed using bootstrapped 95% confidence intervals based on 5,000 resamples.

Significant indirect effects were found for H15c (SP → PT → INT; β = 0.040, p = 0.046), H15d (TMS → PT → INT; β = 0.059, p = 0.037), H15e (FC → PT → INT; β = 0.085, p = 0.021), and H15f. (GSR → PT → INT; β = 0.055, p = 0.023). These findings indicate that security and privacy, top management support, financial capability, government support, and regulations influence adoption intention indirectly through perceived trust.

In contrast, H15a (PC → PT → INT; β = − 0.021, p = 0.431), H15b (PR → PT → INT; β = -0.039, p = 0.149), and H15g (HR → PT → INT; β = 0.033, p = 0.077) were not supported, indicating non-significant indirect effects through perceived trust. These findings indicate that perceived trust mediates selected relationships rather than all relationships in the model. In particular, SP and GSR operate primarily through perceived trust rather than through direct effects.

In contrast, H15g (HR → PT → INT) was not supported (β = 0.033, p = 0.077), indicating a non-significant indirect effect. Although hospital readiness significantly increased perceived trust (H9; β = 0.140, p = 0.025), this effect did not translate into a significant indirect effect on adoption intention. H15a (PC → PT → INT) was also not supported (β = − 0.021, p = 0.431), and H15b (PR → PT → INT) was not supported (β = − 0.039, p = 0.149), indicating that perceived complexity and perceived risk did not influence adoption intention through perceived trust. Relative advantage (H1) was not tested for mediation because it was hypothesized to directly influence adoption intention. Overall, these results indicate that perceived trust mediates selected relationships, with significant indirect effects observed for SP, TMS, FC, and GSR. Complete path coefficients were presented in Table 8.

#### Explanatory and predictive power

The structural model was assessed using R^2^, f^2^, and Q^2^. Predictive relevance was evaluated using Stone-Geisser’s Q^2^^155^. The coefficient of determination (R^2^) indicates the variance in endogenous variables explained by the predictors^156^. Accordingly, R^2^ values were calculated for intention to adopt BCT and perceived trust.

In PLS-SEM, R^2^ values of 0.75, 0.50, and 0.25 are commonly interpreted as substantial, moderate, and weak, respectively, according to Chin. As shown in Table 9, the R^2^ values for perceived trust and intention to adopt BCT are 0.757 and 0.671, respectively, indicating substantial to moderate explanatory power. The R^2^ value of 0.757 for perceived trust indicates that perceived complexity, security and privacy, perceived risk, top management support, hospital readiness, financial capabilities, and government support and regulations explain 75.7% of its variance.

Perceived complexity, security and privacy, relative advantage, perceived risk, financial capability, government support and regulations, and perceived trust explain 67.1% of the variance in intention to adopt blockchain. As shown in Table 9, we have two endogenous variables, with R^2^ values of 0.757 for perceived trust and 0.671 for intention to adopt BCT, indicating substantial to moderate explanatory power. The results reveal that the model accounts for 75.7% of the variance in perceived trust and 67.1% of the variance in blockchain adoption intention. Effect size (f^2^) measures the extent to which each predictor explains the endogenous constructs. According to Cohen’s criteria^139^. The f^2^ values of 0.02, 0.15, and 0.35 indicate minor, medium, and large effects, respectively.

For intention to adopt BCT, relative advantage demonstrated a medium effect (f^2^ = 0.254), followed by perceived risk (f^2^ = 0.073), perceived complexity (f^2^ = 0.056), perceived trust (f^2^ = 0.051), financial capabilities (f^2^ = 0.035), government support (f^2^ = 0.024), and security and privacy (f^2^ = 0.012). For perceived trust, financial capabilities showed a medium effect (f^2^ = 0.138), followed by top management support (f^2^ = 0.124), government support (f^2^ = 0.056), security and privacy (f^2^ = 0.052), hospital readiness (f^2^ = 0.039), perceived risk (f^2^ = 0.028), and perceived complexity (f^2^ = 0.009).

The results show that relative advantage has the largest direct effect on adoption intention, followed by perceived risk, perceived complexity, perceived trust, and financial capability. For perceived trust, financial capability, and top management support show the largest effects. Predictive validity was evaluated using Stone-Geisser’s Q^2^ test, which assesses the model’s ability to predict omitted observations by cross-validation and a blindfolding technique. Q^2^ values exceeding zero indicate predictive relevance, with values above 0.25 considered medium and above 0.50 considered large predictive validity.

As shown in Table 9, the Q^2^ values were 0.721 for perceived trust and 0.662 for intention to adopt blockchain. Both values were well above zero, indicating strong predictive validity. These Q^2^ values indicate strong predictive relevance for the endogenous constructs.

Collinearity refers to how predictor (exogenous) constructs within a model are related to one another^139^. In SmartPLS, the Variance Inflation Factor (VIF) has been used for each predictor to identify collinearity. In this study, all VIF values were under the five thresholds, confirming that the collinearity levels were within the acceptable range.

## Discussion

This study aims to develop and empirically test a model to provide insight into the factors influencing blockchain adoption in public hospitals operating within Egypt’s Universal Health Insurance System (UHIS).

The findings reveal that relative advantage, financial capability, and perceived trust directly influence blockchain adoption intention, while top management support contributes indirectly by strengthening perceived trust. Complexity and risk create barriers. Security, privacy, government support, and regulations did not have significant direct effects on adoption intention. Specifically, H2 (SP → INT) was rejected (β = 0.116, p = 0.216, f^2^ = 0.012), and H12 (GSR → INT) was rejected (β = 0.154, p = 0.070, f^2^ = 0.024). These findings indicate that SP and GSR do not directly predict adoption intention in this context. In line with these results, SP and GSR contribute to adoption by strengthening perceived trust. In this context, SP and GSR influence adoption intention indirectly through perceived trust rather than through significant direct effects. Hospital readiness also builds trust, as H9 (HR → PT) was supported (β = 0.140, p = 0.025, f^2^ = 0.039), but this trust does not significantly translate into adoption intention in this sample. The data show that relative advantage was the strongest observed direct predictor of adoption intention H1 (RA → INT) was supported (β = 0.545, p = 0.001, f^2^ = 0.254). These findings are consistent with previous studies across healthcare, manufacturing firms, and supply chain contexts, where similar determinants have been identified^157^. In these contexts, the perceived benefits must clearly outweigh the implementation costs before adoption proceeds^101,158,159^. One of blockchain’s characteristics is its ability to enable secure and efficient data exchange among multiple stakeholders within UHIS. In the Egyptian context, characterized by regulatory and operational uncertainty, relative advantage serves as a risk-mitigation strategy rather than simply a performance-improvement tool^68^.

Regarding security and privacy, H2 (SP → INT) was rejected (β = 0.116, p = 0.216, f^2^ = 0.012), indicating that security and privacy do not directly influence adoption intention in this sample. Such a result is surprising, as Egypt has data protection obligations under the Personal Data Protection Law^160^. A direct positive relationship was initially expected because the blockchain’s cryptographic architecture helps reduce the health insurance sector’s vulnerability to data breaches. This result aligns with previous studies suggesting that security is a characteristic of blockchain architecture, but it does not, in itself, drive adoption intention. In the Egyptian public healthcare context, security and privacy are often treated as baseline compliance requirements rather than strategic drivers of technology adoption. Hospitals tend to prioritize implementation feasibility, system integration, and resource availability, which may explain why security considerations do not directly translate into adoption intention. Such findings differ from findings reported in government services and the financial sector, where security concerns have been reported to influence adoption decisions in certain contexts^49,116^. In the healthcare sector, the value of adopting blockchain systems lies in operational efficiency gains for hospitals managing insurance systems, not solely in security compliance. Specifically, H3 (SP → PT) was supported (β = 0.151, p = 0.009, f^2^ = 0.052), indicating that security and privacy significantly contribute to building perceived trust. This suggests that while security does not directly motivate adoption decisions, it plays an important role in building confidence in the technology, particularly in environments where concerns about data handling and system reliability remain salient.

Regarding perceived complexity, H4 (PC → INT) was supported (β = − 0.255, p = 0.008, f^2^ = 0.056), indicating that blockchain complexity negatively affects hospitals’ intention to adopt in this sample. A negative relationship between perceived complexity and blockchain adoption intention emerged explicitly in correlational analyses of previous studies. One example of blockchain complexity during implementation is the need for extensive staff training and resources on blockchain systems. In addition, integrating BCT systems across hospitals and insurance companies requires high technical competence, which can increase time and resource requirements, creating uncertainty among decision-makers about adopting blockchain. Public hospitals in Egypt, as government institutions, are resistant to change. They may rely on traditional, centralized business solutions they trust rather than transitioning to an unfamiliar blockchain architecture^26,71,161–163^. Similar barriers have also been reported in other public-sector contexts, where blockchain implementation is constrained by institutional procedures, technical integration challenges, and limited organizational readiness^164^. In general, decision-makers in the healthcare sector in Egypt focus on outcomes and, in most cases, delegate technical tasks to specialists. For that, complexity can reduce adoption intention but does not significantly decrease trust (H5)^109^. While H4 and H5 were framed to test whether complexity affects both intention and trust, the findings reveal a distinction: complexity influences adoption intention directly but does not significantly affect trust. Egyptian public hospital administrators appear to distinguish operational feasibility concerns from their trust in the technology’s value; they may find blockchain difficult to implement while still believing in its potential^114,165^. In contrast, H5 (PC → PT) was rejected (β = -0.087, p = 0.385, f^2^ = 0.009), indicating that complexity creates practical implementation barriers without decreasing confidence in blockchain’s underlying benefits. Such a finding helps explain why complexity operated as a direct adoption barrier rather than a trust-reducing factor in the Egyptian context.

Similarly, H6 (PR → INT) was supported (β = -0.315, p = 0.003, f^2^ = 0.073), indicating that perceived risk reduces adoption intention, and it surpasses complexity (β = -0.255). These results align with previous studies of blockchain across healthcare, SME, construction, cryptocurrency, and supply chain contexts^74,110,166–168^. In contrast, H7 (PR → PT) was rejected (β = − 0.164, p = 0.087, f^2^ = 0.028), indicating that perceived risk does not significantly reduce perceived trust in this sample. In Egypt, perceived risk appears to reflect concerns about implementation rather than distrust of blockchain itself. One possible explanation is that public hospital administrators may recognize blockchain as a trustworthy technology in principle, but still view its implementation as risky because of legacy system integration, unclear operational procedures, limited blockchain expertise, and dependence on centralized government approval. This may explain why perceived risk reduced adoption intention but did not significantly reduce perceived trust. Developing country SMEs face similar adoption barriers, as documented in^169,170^. To mitigate risks arising from legacy system integration in public hospitals, these findings suggest that policymakers may need to reduce operational risks, provide clear regulatory guidance for blockchain implementation, and address sustainability concerns that may further complicate blockchain adoption^28,171^.

Regarding the role of top management support, H8 (TMS → PT) was supported (β = 0.248, p = 0.001, f^2^ = 0.124), indicating that management support significantly strengthens perceived trust in blockchain adoption. This finding is consistent with previous studies on RFID and mobile health adoption, which found that management support increased confidence in new technologies^25,172,173^. Institutional theory suggests that management support builds organizational credibility in technology adoption, while government regulations and professional standards further strengthen adoption-related decisions. Top management support helps facilitate resource allocation and foster the organizational commitment that health insurance providers need before they trust the technology and begin adopting it^174^.

Conversely, with management support, institutional trust grows, and adoption intention tends to strengthen^92^. In institutional theory, top management often acts as a key source of internal pressure that shapes organizational decisions^22,23^. This influence is stronger in Egypt’s hierarchical public hospital structure, where senior management approval often shapes adoption-related decisions^4,71^. Because external regulatory support for blockchain remains limited, internal leadership becomes the primary legitimizing force^4,152^. When senior managers support blockchain adoption, administrators gain confidence to proceed^174^. Limited blockchain knowledge among hospital administrators may increase their reliance on senior management guidance before supporting blockchain implementation^9,164^.

The data show that H9 (HR → PT) was supported (β = 0.140, p = 0.025, f^2^ = 0.039), indicating that hospital readiness builds trust in adopting blockchain. This finding is in line with previous research on blockchain in healthcare, elderly care, SMEs, and medical supply chain management^11,31,174,175^. Conversely, these findings differ from studies in private sector and developed country contexts^91,93,176^, which report weak correlations between institutional readiness and blockchain adoption intention. In those settings, organizations had strong regulatory frameworks and adequate financial resources, making readiness less decisive. In Egypt, public hospitals face different constraints, as centralized governance requires Ministry approval regardless of organizational readiness^3,4,152^.

The Resource-Based View theory shows that hospitals with robust technological infrastructure and dedicated digital transformation budgets are more likely to adopt blockchain^21,81^. The RBV theory predicts that organizational readiness drives adoption, but Egyptian public hospitals present a different scenario. RBV assumes that organizations with infrastructure, skills, and resources adopt new technologies readily^21,177^. Egypt’s digital immaturity complicates this, as even if hospitals are ready, they lack the specialized blockchain infrastructure needed for adoption^118,178^. Even when hospitals possess the necessary capabilities, implementation decisions often require approval from centralized authorities such as the Ministry of Health^4,152^. Low knowledge about blockchain compounds the problem; administrators with adequate infrastructure still hesitate regarding the adoption decision without supportive institutional policies^164^. These findings do not contradict institutional or resource-based theories, but rather reflect how these theoretical mechanisms operate differently under the Egyptian public healthcare context.

Regarding financial capability, H10 (FC → INT) was supported (β = 0.221, p = 0.030, f^2^ = 0.035), indicating that resource availability shapes adoption decisions in its own right, not just through trust. This is consistent with the Egyptian public hospital context, where Ministry of Health budgets are typically limited. The upfront and ongoing costs of blockchain infrastructure, technical staff, and maintenance are difficult to absorb without dedicated funding. This aligns with prior studies identifying financial constraints as key barriers to blockchain adoption in healthcare and resource-constrained environments^12,17,81^.

This suggests that trust alone is insufficient to drive adoption without adequate financial resources. Although RBV theory emphasizes the role of organizational readiness in increasing adoption, the results indicate that financial capability plays a more influential role. Readiness indicates that infrastructure may exist, whereas financial capability reflects actual resource allocation^21,177^. In centralized public healthcare, hospitals with dedicated funding may be better able to initiate projects with fewer administrative delays. Such a pattern explains why financial capability is considered a stronger predictor of adoption than readiness^81^.

Additionally, H11 (FC → PT) was supported (β = 0.355, p = 0.001, f^2^ = 0.138), showing that financial capabilities build trust more powerfully than they directly drive adoption in this sample. The resource-based view theory explains that financial availability increases partners’ trust in the feasibility of implementation and builds stakeholders’ confidence in BCT-based HIS adoption in Egyptian hospitals^81,92^. Accordingly, hospital administrators should treat financial planning as both an operational requirement and a trust signal to partners. While RBV suggests both matter^21,177^, hospitals with dedicated budgets demonstrate actual investment willingness rather than simply the intention to adopt the technology. Many Egyptian public hospitals possess basic technical infrastructure but lack allocated blockchain funding. Financial resources, therefore, reflect institutional commitment, not just resource availability. Such a factor accounts for financial capability’s stronger trust-building effect (β = 0.355) compared to technical readiness in adoption decisions^81^.

Moreover, H12 (GSR → INT) was rejected (β = 0.154, p = 0.070, f^2^ = 0.024), indicating that government support and regulations do not directly drive adoption intention, which was unexpected given that providing regulatory frameworks usually facilitates technology adoption. One plausible explanation is that organizational support creates trust without providing the operational conditions required for adoption, such as adequate budgets, legacy system integration, and cross-sector coordination among the Ministry of Health, insurance providers, and technology vendors^63,179,180^. Government support and regulations primarily contributed to adoption intention indirectly by strengthening perceived trust. In the Egyptian public healthcare context, regulatory support may enhance institutional legitimacy and trust but does not directly translate into adoption decisions, which are more strongly constrained by financial and operational considerations. Specifically, H13 (GSR → PT) was supported (β = 0.198, p = 0.012, f^2^ = 0.056). This result is consistent with previous studies in supply chain and financial sector research^49,63^. The Ministry of Health and data protection regulations provide clear rules on data ownership and liability, building institutional trust in blockchain^107,160^. However, it contrasts with SME studies, where government support showed no significant effect on trust, highlighting that healthcare depends more on regulatory trust than other industries^165^.

Policymakers need to provide practical support through dedicated funding, implementation guidelines, and pilot programs that help hospitals move from intention to implementation. Institutional theory assumes regulations drive blockchain adoption through compliance pressure^22–24^, but blockchain regulatory frameworks in Egypt remain weak, fragmented, and with limited enforcement mechanisms^4,152^. Ministry approval for adoption often comes without funding, infrastructure, or implementation guidance. Limited blockchain knowledge among policymakers generates regulations lacking practical implementation guidance^164^.

Therefore, the results indicate that regulations influence trust (H13) but do not directly lead to actual adoption (H12 not supported)^4,71^. Such findings suggest that, in the Egyptian public healthcare context, institutional legitimacy is not enough without practical implementation support.

For perceived trust, H14 (PT → INT) was supported (β = 0.239, p = 0.006, f^2^ = 0.051), showing a significant positive relationship between perceived trust and intention to adopt blockchain. This finding differs from prior studies^17^, which argue that trust alone does not ensure technology adoption when practical barriers such as infrastructure limitations, integration complexity, and high costs remain. In healthcare contexts, these operational concerns may be more immediate than trust-related issues.

In the Egyptian context, trust helps reduce some concerns related to data privacy, system complexity, and implementation costs. Organizations build trust gradually, starting with blockchain’s security, then its operational reliability, and finally its strategic benefits. Each stage addresses a specific barrier and strengthens adoption intention^92,124,181,182^.

In response to the first research question, the results show that in this sample, relative advantage was the strongest positive factor, where H1 (RA → INT) was supported (β = 0.545, p = 0.001, f^2^ = 0.254). Financial capability influenced adoption both directly, as H10 (FC → INT) was supported (β = 0.221, p = 0.030, f^2^ = 0.035) and indirectly through trust, as H15e (FC → PT → INT) was supported (β = 0.085, p = 0.021), while it also significantly influenced trust, where H11 (FC → PT) was supported (β = 0.355, p = 0.001, f^2^ = 0.138). Top management support, where H8 (TMS → PT) was supported (β = 0.248, p = 0.001, f^2^ = 0.124), and perceived trust, where H14 (PT → INT) was supported (β = 0.239, p = 0.006, f^2^ = 0.051), helped reduce uncertainty. Hospital readiness increased trust, as H9 (HR → PT) was supported (β = 0.140, p = 0.025, f^2^ = 0.039). Two factors acted as barriers: perceived complexity, where H4 (PC → INT) was supported (β = − 0.255, p = 0.008, f^2^ = 0.056), and perceived risk, where H6 (PR → INT) was supported (β = − 0.315, p = 0.003, f^2^ = 0.073), with risk showing a stronger effect in this sample. Security and privacy, where H2 (SP → INT) was rejected (β = 0.116, p = 0.216, f^2^ = 0.012), and government support, where H12 (GSR → INT) was rejected (β = 0.154, p = 0.070, f^2^ = 0.024), did not directly affect adoption. This reinforces that these factors operate primarily through perceived trust rather than through direct effects. Instead, security and privacy first built trust, where H3 (SP → PT) was supported (β = 0.151, p = 0.009, f^2^ = 0.052), which then influenced adoption. Similarly, government support strengthens perceived trust, where H13 (GSR → PT) was supported (β = 0.198, p = 0.012, f^2^ = 0.056), which then influences adoption intention rather than directly driving it. Overall, these findings reinforce that SP and GSR influence adoption primarily through perceived trust rather than through direct effects.

The mediation analysis shows that trust does not mediate the effects of complexity, where H15a (PC → PT → INT) was rejected (β = − 0.021, p = 0.431), perceived risk, where H15b (PR → PT → INT) was rejected (β = − 0.039, p = 0.149), or hospital readiness, where H15g (HR → PT → INT) was rejected (β = 0.033, p = 0.077), confirming that these factors influence adoption separately from trust formation in this setting^108,109^. One plausible explanation is that Egyptian hospital administrators treat complexity and risk as practical implementation problems, not as factors that reduce their confidence in blockchain.

The rejection of H15g presents an important theoretical finding. Hospital readiness builds trust, as H9 (HR → PT) was supported (β = 0.140, p = 0.025, f^2^ = 0.039), but does not translate that trust into adoption intention, where H15g (HR → PT → INT) was rejected (β = 0.033, p = 0.077), indicating that readiness alone is insufficient to drive adoption. According to RBV theory, organizational resources, technological capabilities, financial assets, and human expertise enable the adoption of new technologies^21,177^. However, the findings suggest that this relationship may be weaker in the context of Egyptian public hospitals because low digital maturity undermines the quality of technological readiness^9^. Centralized governance structures prevent hospitals from implementing technologies independently, requiring Ministry of Health authorization regardless of organizational capabilities^3,4^. Limited blockchain literacy among administrators further inhibits translating readiness into confident decisions^164^. Egyptian hospitals highlight a contextual limitation in applying RBV theory where possessing resources does not necessarily mean having the authority to control or use them in hierarchical institutional contexts^21,81,152,177^.

Three interconnected factors can explain this gap. First, the Ministry of Health’s approval process involves complex administrative levels, regardless of the hospital’s readiness. As a result, even well-equipped hospitals must wait for bureaucratic approval before proceeding. Second, insurance organizations focus more on funding and regulatory approval than on whether hospitals are technically ready. Without a clear regulatory framework, adoption cannot proceed even when funding is available. Third, beyond this, budget constraints, incompatibility with legacy systems, and weak cross-sector coordination remain key obstacles that technical readiness alone cannot overcome. Such a difference between H9 and H15g aligns with^75^, which distinguishes technology assessment from actual adoption commitment^11,31^. Prior studies in public-sector contexts confirm similar findings, showing that infrastructure readiness signals capability but does not guarantee technology adoption without complementary financial resources and regulatory support^31,91,183^.

In response to the second research question, the results show that trust mediates four relationships: H15c (SP → PT → INT) was supported (β = 0.040, p = 0.046), H15d (TMS → PT → INT) was supported (β = 0.059, p = 0.037), H15e (FC → PT → INT) was supported (β = 0.085, p = 0.021), and H15f. (GSR → PT → INT) was supported (β = 0.055, p = 0.023). These findings indicate selective mediation rather than full mediation, as several indirect paths (H15a, H15b, H15g) were not supported. Egyptian administrators build trust through institutional approvals, financial commitments, and regulatory support, not through technology assessment alone.

Financial capabilities signal partnership credibility, management endorsement gives blockchain institutional legitimacy, and government frameworks clarify legal ambiguities under Egypt’s personal data protection law^160,177^. These institutional (management, government) and resource (financial) factors appear to mediate through trust because they strengthen perceived legitimacy and credibility^23,81^. These findings address the second research question by showing that perceived trust mediates selected relationships in blockchain adoption.

The findings have some limitations, and the results should be interpreted carefully because the study included only IT staff from six governorates and was conducted in a single survey. Non-IT stakeholders, such as clinicians, may have different priorities or other factors, and results might differ across other governorates or private hospitals. These limitations may affect the generalizability of the findings beyond the Egyptian public healthcare context.

The proposed framework emphasized institutional theory (regulatory pressure, government support, top management) and RBV (readiness, financial capabilities) as central drivers^21–23,177^. However, Egypt faces several contextual barriers. These include weak and fragmented regulatory frameworks^4^, low digital maturity, conservative organizational culture, and inadequate blockchain knowledge among administrators and policymakers^9,71^.

Regulatory pressure may have had limited influence because enforcement mechanisms remain weak. Similarly, hospital readiness may not lead to adoption when hospitals lack the authority to implement blockchain independently. Knowledge gaps among administrators and policymakers may also weaken the effects of these theoretically important factors. Together, these conditions help explain why some factors performed differently than expected in the Egyptian context^9,164^. These findings do not invalidate TOE, RBV, or Institutional Theory; rather, they show that these frameworks may require contextual adjustment when applied to public hospitals in developing-country health insurance systems, such as Egypt^3,4,53,71^.

The findings reflect Egypt’s healthcare environment, where hierarchical governance and limited institutional trust push hospital administrators to focus more on resource-related factors rather than technology assessment^4^. Theoretically, this suggests that the mediating role of trust may depend on the institutional environment rather than on technology characteristics alone^71^.

## Conclusion and implications

This study examined the factors associated with blockchain adoption intention in Egyptian public hospitals operating within UHIS, addressing an important gap in empirical research. The analysis focuses on adoption decisions at the hospital level, while health insurance systems represent the operational context within which these decisions occur. Using an integrated TOE–RBV–Institutional Theory model, the study identified direct and trust-mediated adoption pathways. Perceived trust significantly mediated four of the seven tested relationships. Security and privacy, government support, and regulations did not have significant direct effects on adoption intention; instead, their influence operated indirectly through perceived trust, indicating selective mediation for specific factors.

The results show that, within the study sample, relative advantage is the strongest observed direct predictor of adoption intention, while financial capability influences adoption both directly and through trust. Top management support may reduce adoption hesitancy by increasing confidence in blockchain, while perceived risk and complexity remain the main barriers. Overall, the findings suggest that blockchain adoption in Egyptian public hospitals appears to be shaped more by perceived value, available resources, and institutional trust than by technology features alone.

By integrating TOE, the resource-based view, and institutional theory, this study offers a broader explanation of blockchain adoption in Egyptian public hospitals, while treating trust as a selective mediating factor.

The findings show that mediation effects vary across factors. In this study, perceived trust acts as a selective mediator in relationships involving security and privacy, top management support, financial capability, and government support and regulations. In contrast, complexity and risk influence adoption directly and do not show a significant effect on trust. Hospital readiness increased trust but did not translate into adoption through mediation. These results suggest that trust tends to mediate adoption under supportive institutional conditions such as regulatory clarity and financial resources, rather than due to technology characteristics alone. The findings also indicate that top management support and regulatory frameworks shape public hospital trust in blockchain, providing empirical support for institutional theory in this context.

The findings provide partial support for the resource-based view theory: financial capability serves as an important indicator to stakeholders, building trust that translates into adoption. However, hospital readiness builds trust but does not lead to a significant effect on adoption intention. The model explains 67.1% of adoption intention variance and 75.7% of perceived trust variance, indicating that a multi-theoretical approach captures important adoption dynamics in this context.

These findings suggest that hospitals may benefit from focusing on understanding and communicating the practical benefits of blockchain adoption. The Ministry of Health, insurers, vendors, and hospitals should collaborate to promote these benefits through staff training programs, while blockchain vendors should share real-world success stories from similar organizations in developing countries.

Perceived complexity had a strong negative effect within the study sample, indicating that simplifying system integration and improving usability are likely to be important for facilitating blockchain adoption. Role-specific training for both technical and non-technical staff, combined with workshops and technology trial opportunities, will help administrators assess fit and build confidence before full deployment.

Without stronger collaboration among insurers, hospitals, vendors, and regulators, institutional trust alone may not be sufficient to translate into actual implementation. Policymakers should establish knowledge-sharing platforms to develop common implementation standards and turn institutional trust into actual adoption.

Egyptian public hospitals build trust through management support and regulations, but these factors do not directly drive adoption intention. Instead, hospitals evaluate technology complexity and implementation feasibility separately.

Top management commitment builds trust by legitimizing blockchain adoption and allocating the necessary resources. Administrators should conduct readiness assessments, run pilot programs aligned with Egypt’s Vision 2030, and develop risk mitigation plans that address implementation costs, legacy system integration, and compliance with the Personal Data Protection Law.

The Ministry of Health could go beyond regulatory approval to provide practical implementation guidelines, dedicated funding, and workforce development programs.

The theoretical framework emphasized institutional theory (regulatory pressure, government support, top management) and resource-based factors (hospital readiness, financial capabilities) as central drivers of adoption. However, the Egyptian context produced different outcomes. These contextual barriers, including weak institutions, limited digital infrastructure, and knowledge gaps, help explain why theoretically central factors operated differently than predicted in this study. These limitations should be considered when interpreting the generalizability of the findings.

Finally, clearer laws and regulations are needed to further support the implementation of blockchain in healthcare. Egypt should set standards for encrypting patient data, managing claims through smart contracts, and licensing blockchain service providers. These regulations should include audit and compliance processes, dispute resolution mechanisms for health insurance systems, and interoperability guidelines to ensure blockchain works safely and effectively across Egypt’s healthcare sectors.

### Limitations and future work

This study examines public hospitals’ intention to adopt blockchain rather than actual use, because the technology is still at an early stage. This distinction may limit the interpretation of adoption outcomes, particularly as some factors (e.g., security and privacy and government support) operate indirectly through perceived trust rather than directly influencing adoption intention.

The sample was limited to IT staff, potentially introducing response bias, as non-IT stakeholders like clinicians may exhibit different attitudes toward blockchain adoption. Additionally, the focus on six governorates in the early phase of Egypt’s Universal Health Insurance System limits generalizability to all 27 governorates or private hospitals. The cross-sectional design limits causal interpretation because relationships are based on correlations, and longitudinal studies could validate these relationships over time. Future research should examine whether the observed trust-mediated adoption pattern remains stable when hospitals move from adoption intention to actual blockchain implementation. These limitations should be considered when interpreting the generalizability of the findings beyond the Egyptian public healthcare context.

Future research should include a wider range of healthcare professionals to develop a multilevel model that captures both organizational decisions and individual user perspectives, allowing for a deeper examination of how perceived trust mediates adoption decisions across different stakeholder groups. This study focused exclusively on public-sector hospitals and did not include Egypt’s private-sector hospitals. Comparing public and private Egyptian hospitals would show how differences in funding structures and governance conditions produce different adoption pathways.

Extending this research to other countries requires accounting for cultural, legal, and economic differences, and multi-country studies across developing healthcare systems would help assess whether the trust-mediation pattern found here applies more broadly.

Future studies could examine whether specific interventions, pilot programs, training initiatives, and government funding help translate institutional trust into blockchain adoption. Future research should also further investigate factors such as security and privacy and government support, which in this study influenced adoption indirectly through perceived trust rather than through direct effects.

Data privacy remains an important challenge. Open data sharing can expose patient information, and permanent records need careful protection to prevent unauthorized access. Compliance with Egypt’s Personal Data Protection Law requires clear rules on data ownership, sharing, and legal liability.

Future studies could also examine collaborative financing models in which public hospitals, private hospitals, and health insurance providers share implementation costs for blockchain infrastructure.

Tools such as data encryption, access control, and anonymization are important to protect patient privacy while preserving blockchain’s advantages. Future research should examine how privacy concerns affect adoption intentions in Egyptian public hospitals and how blockchain systems can be designed to protect patient data, control access, and comply with regulatory requirements. This would provide practical guidance on how organizations can balance data sharing and patient privacy in blockchain-based health insurance systems.

## Data Availability

The datasets generated and/or analyzed during the current study are not publicly available due to institutional and ethical restrictions related to the Universal Health Insurance Authority. The data are available from the corresponding author upon reasonable request and with permission from the Universal Health Insurance Authority.
